# Observations on Those Diseases of Females Which Are Attended by Discharges; Illustrated by Copper-Plates

**Published:** 1816-06

**Authors:** 


*Observations on those Diseases of Females which are attended by Discharges; illustrated by copper-plates.* By Charles Mansfield Clarke, &c. &c. Part I. *Mucous Discharges*. pp. 304. London.

( Continued from our last Number, page 413. )

### Carcinoma Uteri.

In the commencement of Carcinoma Recti and C. Uteri, the two diseases may be distinguished; but in the latter stages, the surrounding parts are drawn into disease in both cases, and both are equally fatal.

Scirrhous enlargements of the uterus will sometimes go to a great extent without ulceration. The hard tumor rising from the cervix uteri, and hard thickening of the cervix itself, both disposed to ulcerate, are the subjects of the present chapters.

Carcinoma uteri is much more common than C. recti, attacking principally middle-aged women, with symptoms not very violent at first, but gradually increasing. By C. uteri, Mr. Clarke means a tumor near to, or a thickening of, the cervix uteri, disposed to ulcerate. The disease at first attacks *only* the cervix; all other tumors, however hard in their texture, are of a different character, and have a different termination—they do not ulcerate. Real scirrhous tumors seldom become large. Two varieties of this disease are distinguishable at an early stage.—1st var. A firm tumor of a rounded form, springing from the surface of the cervix uteri, or imbedded in it, the other parts of the uterus (excepting a thickening of the parietes) being perfectly healthy. 2d. var. Enlargement and induration of the whole cervix uteri, in structure carcinomatous. In the first variety, there will be some symptoms from mechanical pressure of the tumor on neighbouring parts. In the second variety, these will not take place; because the carcinomatous thickening will rarely acquire a sufficient size to produce them. In women of temperate habits, the disease may go on for a long time without occasioning much inconvenience. But in real carcinomatous tumors of the uterus, the progress is very rapid when any violence is offered them, as in labour. A sense of weight in the vagina is equivocal, as it is felt in all moveable tumors of the pelvis, when they become large. A mucous discharge is a very constant attendant, and checks the progress of the complaint, by unloading the vessels of the womb. This discharge is sometimes tinged with blood, at others, blood itself comes away in great quantities; during which, the tumor generally remains stationary. Profuse evacuations of this kind sometimes end in dropsy, which terminates in death. In C. uteri, if menstruation has not ceased, it becomes irregular and profuse. Dysury is of rare occurrence here; but strangury, from sympathy, is frequent, with mucous secretion from the bladder. The pain counterfeits that attending the passage of a stone from the kidney to the bladder, and is sometimes accompanied by urticaria, and cardialgia. If, in these cases, tonics, alkalies, bitters, aromatics, or particularly mercury, be given, the original disease will probably be increased; hence the necessity of an examination, or minute inquiry, when the foregoing symptoms are observed.

The os uteri, in these cases, generally appears larger than usual, so as to admit the point of the finger, which feels as if surrounded by a firm ring.

*Treatment.* If the system is plethoric, blood should be taken from the arm proportioned to the circumstances of the case. The same may be done from the hypogastrium, or loins, by leeches, or cupping, repeated from time to time on any increase of uneasiness. Blood is more easily taken from the back than from the abdomen. Saline purgatives, largely diluted, should never be omitted. Phos. sodæ will often agree, when all others irritate the stomach. The secretion from the vagina should never be checked, but washed away occasionally by injections of *tepid* water, neither so hot as to increase the vascular action of the vessels; nor so cold as to stop the secretion: from 86° to 94°. Warm clothing, as determining the blood to the surface, is useful; and as lessening the chance of visceral inflammation from atmospheric changes. All local stimuli, sexual intercourse included, are improper. The antiphlogistic regimen, in all its strictness, to be pursued.

Urticaria being a frequent attendant, and as the *febrile* state of the system during this eruption *is detrimental* to the local affection, it should be paid attention to. The bowels should be emptied by a purgative of rhubarb, magnesia, compound spirit of ammonia, and mint water, succeeded by a light bitter infusion thrice a day; to which may be added some carbonate of soda, potash, or aromatic water. Soda water for common drink. Here vegetables disagree, and small quantities of light animal food may be allowed.

If discharges of blood be moderate, they should not be checked; if profuse, the recumbent posture, cold, and local astringents are proper. Preparations of iron are injurious, and always increase the pain and discharge. No cure can, of course, be expected.

### Chap. XVI. Polypus of the Uterus.

Polypus Uteri is an insensible tumor attached to the internal part of this viscus by a small neck. These tumors are very various in respect to size, shape, hardness, &c. The neck of the tumor, surrounded by the os uteri, is contracted, while its base dilates in the yielding vagina. The soft polypus is by no means so common as the hard kind.

*Symptoms.* First. A mucous discharge, in considerable quantity, tinged occasionally with blood, and often inducing great debility before the nature of the disease is suspected. Instead of mucus, coagula of blood are sometimes voided; at others, pieces of a ring like form, from obvious causes. Great fœtor sometimes takes place from the blood remaining in the vagina, inducing a belief that cancer exists; which opinion is strengthened by the sickness of stomach attending the disease. Sense of pressure and bearing down, with pain in the back and groin, are felt in this disease. If the tumor be large, it may press both on the rectum and meatus urinarius obstructing the evacuation of fæces and urine; but such instances are uncommon. Thus gastric irritability, increased secretion of mucus, hæmorrhage, and derangement of the digestive powers, all tend to produce great debility in this complaint. But the true character of the disease can only be ascertained by examination. The only diseases for which polypus may be mistaken, are inversio uteri, and the cauliflower excrescence. The history of the case, and the *insensibility* will distinguish it from the former. The irregularity of surface, origination from the substance of the os uteri, broad base, and watery discharge, attending cauliflower excrescence, will distinguish it from polypus. There is, however, a disease, consisting of a tumor which is insensible, unequal and ragged in surface, coming down from the cavity of the womb into the vagina, surrounded by the os uteri, but without a narrow neck, which may be taken for polypus. Upon an accurate examination, this tumor will be found to be made up of a number of irregular portions which lie parallel with each other. All the symptoms of polypus uteri attend this complaint, and it ultimately proves fatal. M. Herbiniaux, a surgeon at Brussels, has given a good description of this tumor.

Polypus uteri is only to be cured by an operation, which is the application of a ligature round its neck. The prognostic is, in general, favourable.

### Fleshy Tubercle of the Uterus.

This is a hard, whitish tumor, situated sometimes upon the surface of the uterus, between the muscular and peritoneal coat, sometimes projecting into the cavity of the uterus, and occasionally imbedded in its substance. There is sometimes more than one tumor; their forms various; commonly spherical or hemispherical.—When projecting into the cavity of the uterus, their surface is smooth.—When on the exterior of that organ, they are otherwise. These tumors are sometimes not larger than a pea; sometimes they weigh several pounds, and occupy a great part of the abdominal cavity. The fleshy tubercle has been mistaken for ovarian dropsy and pregnancy. The first is an error of little importance, as the symptoms in both are mechanical, and do not interfere with life, unless by pressing on contiguous parts. The fleshy tubercle of the uterus is much more resisting than the cyst of an ovarian dropsy. In the latter, fluctuation may be felt by striking the abdomen gently with the hand. From pregnancy, it is sure to be distinguished, after a few months, by the motion of the child in the former. It is not an uncommon disease—occurs in the married and unmarried, but rarely before the twentieth year. It has no disposition to ulcerate, or suppurate; but inflammation is apt to occur in the neighbouring parts. Nothing is known respecting its cause.

*Symptoms.* An increased discharge of transparent mucus sometimes attends. The earlier symptoms are frequent disposition to make water, and to empty the rectum: retroversio uteri, and suppression of urine may occur in this disease. Cramp in one or both legs; œdema of one or both feet. When the tumor becomes large, there may be difficulty of passing the fæces, and total inability of emptying the bladder; bearing down, not relieved by the horizontal posture. On examination, a large, hard, resisting tumor will be felt; but the os uteri will have undergone no change—will not gape as in carcinoma; nor will pain be complained of when the tumor is pressed upon. The constitution is seldom affected; and when it is so, it is merely from pressure. It is totally uninfluenced by medicines internally or externally applied. The occasional symptoms only can be attended to with any advantage.

The XVIIIth. On verrucæ, or warty tumors, arising from the vestibulum, contains nothing very new or very interesting.

The two last Chapters in this work, XXI. and XXII. are interesting.—Viz. On the transparent mucous discharge from the vagina, unaccompanied by any organic derangement of the sexual organs.

There are two distinct and dissimilar classes of these discharges:—one arising from increased action of the vessels of the parts; the other from debility, in which the former, indeed, may end. Those discharges are, however, much more frequently from debility than increased action.

### From increased Action.

Women of naturally plethoric habits, possessing great strength of constitution, are more liable to this than weaker women. Females, who, in the middle of life, indulge much in the pleasures of the table, especially in wine or spirits—whose habits of life are sedentary, and who take little exercise in the open air, are liable to become suddenly corpulent, and form too much blood, as may be seen by the pulse and superficial veins. These women are in reality weak, though apparently strong—they are easily fatigued, and devoid of energy. In many of these, a slow enlargement of the liver takes place, as may be ascertained by the hand, while little bile is secreted, and the stools are clayey or white, with much fœtor of putrescency. Constipation and obesity increase; while a vaginal discharge takes place, and the menstrual flow is augmented. Upon inquiry, giddiness, and somnolency will be complained of, with head-ache, perhaps indistinct vision, or the appearance of sparks before the eyes. Many years may elapse in this way, without much apprehension of danger, the symptoms being occasionally relieved by a spontaneous hæmorrhage from the nose, or other natural or artificial evacuation; when, all at once, the woman shall be attacked with apoplexy, or some great internal hæmorrhage that quickly terminates the scene! Or she may gradually become dropsical, and die that way. The mucous discharge is, in these cases, probably, an effort of nature to relieve the vascular plethora of the system; and hence, when injudiciously checked, without substituting other evacuations, the violence of the symptoms is increased.

Mr. C. has examined the bodies of females dying during the prevalence of these symptoms, and has found the uterus somewhat, but not much enlarged; while the liver was often increased to twice its natural size, with a uniform density and yellowness, but no other particular morbid alteration of structure.

"The objects in the treatment of this case are, to unload the vessels, by removing at once a large quantity of blood; to prevent its too quick formation in future; to restore, if possible, the liver to a healthy state: afterwards to moderate the vaginal discharge, or to diminish the inconveniences attending its continuance; and, lastly, to lay down proper rules for the patient's conduct, in order to prevent a return of the symptoms."

To obviate local fulness, if such be present, cupping glasses between the shoulders; to the lower part of the abdomen; to the loins; to the region of the liver. If no such local symptoms be distinguishable, venæsection; saline purgatives in small doses thrice a day; antiphlogistic regimen.

Exercise is to be gradually increased; fermented and spirituous liquors to be prohibited; and if their use had been long continued, with gastric debility, spices, aromatic seeds, and ammonia, may supersede them. Until the plethoric state of the system be reduced, tepid water *alone* is to be injected into the vagina; afterwards weak solutions of sulphate of zinc. Local increased action of the vessels, as frequent sexual intercourse, may give rise to the disorder, and is to be obviated if possible. After local or specific inflammation of the vagina, with purulent secretion, leucorrhœa sometimes succeeds, and is very difficult of cure.

In the treatment of transparent mucous discharges from the vagina, produced by increased action of the vessels, local remedies will be principally required, consisting of abstractions of blood by leeches, scarificators to the lower parts of the abdomen, or to the back, repeated when necessary. The bowels to be kept open by mild purgatives. The food to be simple and light, avoiding spices and liquors. Sexual intercourse to be prohibited. Cold solutions to the parts, (liq. plumbi acet. c.) and afterwards, when the secretion continues from relaxation of the membranes, astringent washes.

The last chapter in the work, On transparent mucous Discharge depending upon Debility, is short but interesting. We shall give it nearly in the words of the author.

" That women, whose vagina has lost its tone, become liable to this disease, has been before remarked in that part of this work where some general observations upon the nature of the vaginal discharge were made. Whatever tends to produce debility of the system, may lay the foundation of this complaint; such as long diseases, profuse hæmorrhages, or anxiety of mind.

" Women who live in a moist atmosphere, who keep bad hours, who spend much of their time in bed, or who inhabit hot rooms (being generally weak women, and having a relaxed vagina), will be apt to be affected by the complaint.

" It sometimes arises in women who suckle their children for too long a time, and it will often subside spontaneously upon the child being weaned.

" The quantity of discharge, and also its consistence, is very various in different cases. It sometimes comes away in a liquid form; at other times it is ropy.

" A pain in the back attends many cases of the disease. This symptom is, however, frequently found where great debility of the system is present.

" The continued drain from the system increases the original weakness; and the quantity of blood remaining is by degrees so much reduced, that the surface of the body becomes every day paler, till at length the cutaneous vessels are completely emptied of their contents; and at this time the skin assumes an appearance resembling that of a dead body. The colour of the sebaceous glands of the skin is evident through the cuticle; so that to the paleness of the skin is superadded an appearance of yellowness, which is not the effect of absorption of bile, for the urine will be found clear and colourless, and the tunica sclerotica of the eye will retain its pearl-coloured appearance. The exact balance between the secreting arteries and the absorbents being destroyed, the cellular membrane becomes filled with fluid, and the integuments acquire a doughy look and feel. This fluid effused, pervades the cells of the cellular membrane throughout the body; the legs and feet swell towards night, and in the morning this swelling subsides, and the face becomes puffed; a shortness of breathing succeeds, which is increased by the horizontal posture, and is rendered most distressing when the patient is going up an ascent, or endeavours to read aloud. Violent palpitations of the heart occasionally give the woman great uneasiness; and this symptom sometimes increases to so considerable a degree, that the action of the heart may be heard by a by-stander. During the continuance of these palpitations, the patient becomes very faint, and often considers herself to be dying.

"The circulation in the extremities is very languid, and the hands and feet are almost always cold; the pulse is feeble, sometimes very quick. The digestive organs not only partake of the general debility, but have more than their proportion of weakness. The appetite for food is lost; the power of digestion is diminished; and from the spontaneous changes which the food undergoes in its passage through the stomach and intestines, the patient becomes much annoyed by flatulence.

"Bile is secreted very irregularly, and sometimes this secretion is suspended. Costiveness is a general attendant on this state of disease. In the farther progress of the case, hectic fever comes on, the difficulty of breathing becomes more extreme, and the patient dies with the symptoms of water in the chest. Although these symptoms are of the most formidable kind, and threaten the life of the patient, they frequently yield to the employment of proper means, which must be directed with skill, pursued with energy, and continued with patience.

"The first care of the practitioner should be to remove, if possible, the causes of the disease. If the patient has been living in a moist unhealthy situation, she should be removed to one which is more dry and salubrious: without attention to this very important circumstance, all the resources of art will be useless.

"It has been stated, upon good authority, that the disease is frequent in Holland, and that it is epidemic in wet autumns. The author has repeatedly seen the complaint attended by the worst symptoms in women who live in damp situations, and in the crowded parts of London, in whom they have quickly disappeared, upon a removal to a more healthy spot. The woman should neither be permitted to breathe a confined air. When the weather permits, she should go out; and the apartments in which she lives and sleeps should be large and well ventilated. Her habits of life should be regular. She should rise betimes in the morning, and retire to rest early in the evening. If she is too weak to sit up the whole of the day, a sofa is to be preferred to a bed.

"Persons who are very weak are much disposed to sleep. This prevents that exhaustion of the powers of the frame which would otherwise take place. Exercise, proportioned to the strength and to the means of the patient, should be recommended: when the weather permits, it should be taken in the open air. Exercise in an easy carriage is preferable to walking, and that taken on horseback to both. The chamber-horse, or elastic plank, may be employed when exercise out of doors cannot be used.

"The food should be of the lightest kind, such as animal broths and jellies, vegetable jellies, bread properly fermented and well baked. It will be better that the patient should not eat solid animal food until the powers of the stomach are in some degree restored, lest fever should be excited by it. When the powers of the digestive organs become more vigorous, recourse may be had to animal food; which should be taken in small quantities, once only in the twenty-four hours, and in the middle of the day. Tender meats, and such as will not be likely to disagree with the stomach, should be selected. Wine, either pure or mixed with water, as may best suit the palate or the stomach, may be allowed in moderate quantities. All wines which have not undergone a complete fermentation will give occasion to flatulency, and are therefore improper.

"The medical treatment is to consist of the employment of means to invigorate the stomach and the constitution, and to restrain the vaginal discharge, which, as long as it continues, must increase the debility.

"If steel and the other metallic tonics are exhibited in the first instance, they will either be rejected, or they will increase the frequency of the pulse and the general heat of the body; and thus, by wearing out its powers, exhaust that strength which they were intended to augment.

"Instead of this plan, the patient should take, two or three times in the day, a draught of infusion of columba, or some other light bitter, with the addition of a few grains of carbonate of ammonia.

"At the end of two or three weeks, it is to be expected that the stomach will have received an increase of strength; and then, instead of the volatile alkali, fifteen or twenty drops of tincture of muriate of iron, or of ammoniated iron, may be added to the draught.

"Sulphuric acid is a useful tonic in these cases, and may be given in an infusion or decoction of bark.

"A mixture of myrrh, steel, and alkali, with some aromatic water in some cases where the stomach will bear it, becomes a valuable remedy.

"Under a continuance of these or similar means, the patient's health will be gradually re-established.

"Care is to be taken to regulate the functions of the bowels until they have acquired sufficient strength to carry down their contents without assistance: for this purpose, the pil. ex aloë c. myrrh. or the pil. gamb. comp. of the last Pharmacopœia, are well enough adapted.

"It is advantageous in some cases, where the biliary secretion is sluggish, to exhibit occasionally a few grains of pil. hydrarg. at bed-time; and in the morning following a rhubarb draught. The strengthening remedies will produce a better effect when this is attended to than when it is omitted.

"A solution of sulphate of zinc or of alum may be thrown into the vagina by a syringe, three or four times a day; and if these should not be sufficiently powerful, such injections must be employed as possess a greater degree of astringency.

"Cold sea-bathing will be found very useful, when no symptoms are present forbidding it; the system, however, should have first rallied a little. Those patients who cannot bear the shock of a cold bath, will occasionally derive great advantage from a shower bath. The temperature of the water may be raised to sixty or seventy degrees, if the cold water d es not agree with the patient."

We have thus presented our readers with a clear view and extended analysis of Mr. Clarke's volume. The matter speaks for itself, and the execution is highly creditable to the talents of our author. In it we recognise the genius of a quondam fellow-student, and call to mind, with reflected pleasure, the many hours we have spent together in the ardour of research——

——————when life itself was new,
And the heart promis'd what the fancy drew.

